# O053. Interictal cerebral posterior circulation in migraineurs with aura: a trancranial color-coded duplexsonography pilot study

**DOI:** 10.1186/1129-2377-16-S1-A159

**Published:** 2015-09-28

**Authors:** Antonio Siniscalchi, Luca Gallelli, Rosario Iannacchero

**Affiliations:** Department Neurology, Coordinator DAMA (Disabled Advanced Medical Assistance), “Annunziata” Hospital, Cosenza, Italy; Department of Health Science, School of Medicine, University of Catanzaro, Clinical Pharmacology and Pharmacovigilance Unit, Mater Domini University Hospital, Catanzaro, Italy; Department of Neurology, Regional Headache Research Center, “Pugliese-Ciaccio” Hospital, Catanzaro, Italy

## Background

Vascular cerebral dysfunction restricted to the posterior circulation in migraineurs has been reported. In this preliminary study we compared mean flow diastolic velocity in the posterior cerebral artery during visual stimulation in interictal state headache-free subjects and migraineurs with aura.

## Matherials and methods

Trancranial color-coded duplexsonography was used to assess mean flow diastolic and systolic velocity changes in the posterior cerebral artery, in particular P2, during visual stimulation.

## Results

An increase in vascular reactivity of mean flow diastolic velocity was observed during visual stimulation in headache-free subjects (Group 1, 10 patients) and in the patients affected by migraine with aura (Group 2, 7 patients), without any differences between right and left sides. Reactivity of mean flow diastolic velocity to visual stimulation was found significantly higher in Group 2 (mean ±SD, 42%±2.6%) vs Group 2 (mean SD, 33.6%±1.8%) (p < 0.001). In contrast, no differences in flow systolic velocity between the two groups were observed.

## Conclusions

These results indicate that occipital cortex is involved in migraineur patients with aura. Moreover, these patients exhibit a larger cerebrovascular response during visual stimulation compared to headache-free subjects.

Written informed consent to publish was obtained from the patient(s).

